# Pleasures and Perplexities of Dental Practice

**Published:** 1865-08

**Authors:** M. L. Jackson


### PLEASURES AND PERPLEXITIES OF DENTAL PRACTICE.

BY M. L. JACKSON.

*Gentlemen of the Iowa Dental Association:*

It has fallen to my lot to write upon the pleasures and perplexities of the Dental practice; and in doing so, I should endeavor to deal simply with truth; dealing with facts that fall under the immediate observation of every dentist in his daily practice.

And let those, who, upon entering the profession with the expectation of having an easy life, be undeceived; for there is no profession requiring so much patience, fortitude, forbearance, and in a word, so much manhood, as Dentistry.

In writing upon this topic, I propose to notice the fair side of the subject first; and in doing so, allow me to say that I consider Dentistry as a profession, one of the noblest callings of man.

To supply the loss of the teeth, the most beautiful organs of man, and restore the features back again to what God intended them to be, is certainly a noble work. "Happily for suffering humanity those beautiful organs can be replaced with artificial ones so closely resembling those planted in the jaw by the hand of nature as to be readily mistaken for such even by the most critical observer."

What dentist has not had his heart throb with a thrill of delight as he has seen the wrinkled, shrunken, haggard, careworn face, restored to a rounded, smooth and beautiful smiling countenance—a transformation from age to youth—deformity to beauty—ill-health to vigor of life. How the heart and tongue of the patient will bless you, and her eyes will beam a thank you, whenever you encounter them; you are her hero, her champion, she dreams of you by night, and thinks and talks your praise in every circle. Nothing can exceed her gratitude.

And then to be able to save those useful organs from destruction and loss—like the skillful physician who applies the remedy to arrest the disease, and restores to life and friends and society a valued friend, We apply the remedy and restore the aching and diseased tooth to one of usefulness and beauty. How often have we been thanked for the restoration of an aching tooth to one of usefulness—how often have we been blessed and almost loaded down with caresses as we have removed the last old offending fang from a mouth redolent with poisonous odors and almost putrid with disease.

In irregularities of children's teeth, how many mothers spend anxious hours about the shape of her darling's mouth, as his teeth begin to assume an irregular circle, they continue to grow more ungainly, the mouth becomes ugly and repulsive to look at—she is told the dentist can remedy the defect: she calls upon you, and by a simple appliance, in many cases, the symetry of the once distorted and unshapen mouth is restored. Will that mother ever forget you in her prayers?

We will now notice some of the perplexities of the practice. It is exceedingly important that every member of a profession should preserve his professional character, and the reputation of his chosen profession. To do this, he must have a remunerative fee for his services as a professional man, and not compromise himself by competing with the huxtering gypsies who infest our country, carrying their office in their carpet bag. Nothing has so great a tendency to degrade a profession, as this guerilla practicing through the country—nothing so disgusts a man of fine feelings as to have a patient come to him and say that he can have his work done at his own house and upon his own terms.

'Tis a misfortune that the masses are not a thinking people, and are readily imposed upon. And should a doctor of not less than a three month term in some dentist's office come along, hang out his shingle, warrant his work, extract teeth without pain, commiserate the people upon the prices other dentists have extorted from them, and join some popular church, he is very apt to run into a practice with the unsuspecting.

Sit yourself back on your dignity, and resolve to do nothing for those only who can appreciate your work, and you will soon find your children engaged in the laudable avocation of begging their bread.

You are frequently sorely tried in a different manner, and by a different class; and it is lucky for the dentist that so few of this class exist.

I refer to females who have had their lives soured by having their conjugal prospects blighted. They will tell you exactly how they want their teeth made; they don't want them to look so artificial; they want them a little uneven, a little irregular; they want them nice and white. You go to work to please them; the teeth are put in; they fit nicely, she is highly pleased with them; all goes as smoothly as a marriage bell, until she meets one of her peculiar class, who happens to be in one of her sour moods; a defect is at once discovered, one side of the teeth are too long. She comes back to you, and the first intimation you have of her presence in the office is a long and dismal sigh; you are startled at the length of her face, and inquire if any friends are dead. She shakes her head mournfully, and says no, it is worse than that, my teeth don't suit me, they are too long, or too short, or too dark, or too white. You reason with her and overcome her objections to this one point. She goes away partially convinced that her friend was wrong. Meets friend No. 2, who has seen No. 1. No. 2 repeats what No. 1 says, and discovers that an eye tooth is a little longer than a lateral incisor. This is a new defect; patient returns with fresh trouble; you again reason with her; she again leaves, but somewhat demoralized. She sees a set you put in for some one else that just suits her; she comes back again thoroughly demoralized; charges you with partiality, claims to be as good as Mrs. A, thinks you might do as good a job for her money as for Mrs. so-and-so. You stand bewildered and befoged, not knowing what to say or do, and just in the midst of this you hear a pull at the bell, glad to escape you rush to the door and meet a little timid girl with something wrapped in paper, who pleasantly says, "Ma wants you to fix her teeth this evening." You take them; a pet job, one you have labored hard to bring to perfection, a set you were proud of; the old story—Ma was cleaning them and dropped them on the hearth; result—the gums in both front blocks broken. And while you thus stand contemplating the frailty of human nature generally, and of artificial teeth particularly, there is another pull at the bell; you invite them to a seat, they decline to take one, but stare you in the face, and ask if you can put a set of teeth in for them without taking an impression. And upon your answering them in the negative, they tell you you cannot make teeth for them then, and leave, thinking teeth a humbug. But you are not done yet. In walks a big burly Irishman, who informs you that he has a small mather of a job for you. His darlint biddy (may the houly angels preserve her) was milking the cow (the ould murthering blackguard, may the fould fiend fly away wid her) the other evening, when she kicked me darlint right in the mouth, and almost killed her intirely; and would you be so kind as to go wid your pullers and pull a couple or three teeth for her. I live just down by the depot —distance only two miles, time 6½ o'clock. Your supper has been waiting an hour or more on you. But you must go, or Irishman will go away mad.

And thus you are often besieged from morning till night with perplexities that would bring sweat to the brow of an Egyptian mummy, and lead you to exclaim in the language of the German poet, "Mine Cod, vat a peoples.

Thus, gentlemen, I have attempted to give you a brief outline of some of the pleasures and perplexities of dental practice, and I am satisfied you all know what I have written is true; and, as I said before, there is no profession requiring so much patience as ours. And the man, to be a successful practioner, must be a man who can adapt himself to all kinds of society. He must be prepared to meet the wants of the poor, as well as those of the rich; attend the wants of the petulant, as well as those of the pleasant; he must meet flattery upon the one hand, and abuse upon the other; he must be courteous and affable, patient and long suffering; and in a word, must be a MAN.

